# A Systematic Analysis of the Relationship of *CDH13* Promoter Methylation and Breast Cancer Risk and Prognosis

**DOI:** 10.1371/journal.pone.0149185

**Published:** 2016-05-06

**Authors:** Jingyu Yang, Heng Niu, Yingze Huang, Kunxian Yang

**Affiliations:** 1 Chest surgery, the First People's Hospital of Yunnan Province, Panlong Campus, 157 Jinbi Road, Kunming, Yunnan, 650000, P.R.C; 2 Medical Faculty, Kunming University of Science and Technology, Kunming, Yunnan 650500, P.R.C; University of North Carolina School of Medicine, UNITED STATES

## Abstract

**Background:**

*CDH13* (cadherin 13) is a special cadherin cell adhesion molecule, and the methylation of its promoter causes inactivation in a considerable number of human cancers. To explore the association between *CDH13* promoter methylation and breast cancer risk and prognosis, we systematically integrated published articles to investigate the diagnostic performance of the *CDH13* methylation test for breast cancer. An independent DNA methylation microarray dataset from The Cancer Genome Atlas project (TCGA) project was used to validate the results of the meta-analysis.

**Methods:**

The relevant literature was searched using the PubMed, Cochrane Library, Web of Science and Google Scholar databases for articles published in English up to May 2015. Data were analyzed using random effect or fixed effect models. The effect sizes were estimated by measuring an odds ratio (OR) or hazard ratio (HR) with a 95% confidence interval (CI). A chi-squared based Q test and sensitivity analysis were performed to examine the between-study heterogeneity and the contribution of single studies to the final results, respectively. Funnel plots were constructed to evaluate publication bias.

**Results:**

Seven hundred and twenty-six breast tumor samples and 422 controls were collected from 13 published studies. The data from the TCGA set include both tumor and normal samples. A significant association was observed between *CDH13* promoter methylation and breast cancer, with an aggregated OR equal to 13.73 (95%CI: 8.09~23.31, z = 9.70, p<0.0001) as measured using the fixed effect model and 14.23 (95%CI: 5.06~40.05, z = 5.03, p<0.0001) as measured using a random effect model. The HR values were calculated as 0.77 (95%CI: 0.27~2.21, z = -0.49, p = 0.622) and 0.38 (95%CI: 0.09~1.69, z = -1.27, p = 0.20) for overall survival (OS) and disease-free survival (DFS), respectively, using the random effect model. This result indicated that breast cancer patients with *CDH13* promoter methylation correlated non-significantly with prognosis and is therefore similar to the findings of the TCGA project.

**Conclusions:**

The methylation status of *CDH13* promoter was strongly associated with breast cancer risk. However, *CDH13* promoter methylation was not significantly related to the OS and DFS of breast cancer and may have limited prognostic value for breast cancer patients.

## Introduction

Breast cancer is the most commonly diagnosed cancer and the leading cause of cancer death in women worldwide. Globally, there were an estimated 1.7 million new cases (25% of all cancers in women) and 0.5 million cancer deaths (15% of all cancer deaths in women) in 2012 [1]. More than 25% of breast cancer patients develop metastatic disease, which is largely incurable and often limited to palliative therapeutic options [2]. The bottleneck in improving survival is therefore early detection [3]. Because DNA hypermethylation is an important mechanism for tumor suppressor gene inactivation in cancer, the measurement of such methylation could act as a powerful biomarker for the early detection of breast cancer. Therefore, we believe that the measurement of DNA methylation could become a powerful means of breast cancer diagnosis.

The cadherin 13 (*CDH13)* gene, a new member of the cadherin superfamily, was isolated recently and has been mapped to 16q24 [4]. Cadherins are transmembrane glycoproteins expressed on the epithelial cell surface that mediate intercellular Ca2+-dependent adhesion, which is important for maintaining normal tissue structure. Abnormalities in the *CDH13* gene have been identified in human malignancies [5, 6]. Moreover, an association between the abnormal expression of *CDH13* and its promoter methylation in lung cancer has been demonstrated [7–9]. However, the diagnostic role of the methylation status of the *CDH13* gene in breast cancer lacks quantitative assessment.

Although *CDH13* methylation in breast cancer has been reported as an effective biomarker for diagnosis [10, 11], the results differ dramatically between different research studies. Here, we conducted a meta-analysis of the risk and prognosis of *CDH13* methylation in relation to breast cancer diagnosis. As we all know, The Cancer Genome Atlas project (TCGA) includes comprehensive clinical and demographic information and has collected hundreds of whole genome DNA methylation microarray datasets of breast cancer samples. Therefore, it provides an additional resource that might be without publication bias. Considering the above factors, we integrated the data from published articles with the data from the TCGA project to evaluate the relationship between *CDH13* promoter methylation and breast cancer. Therefore, an integrated analysis with unbiased conclusions was conducted to examine the relationship between *CDH13* methylation and breast cancer.

## Methods

### Search strategy, selection of studies and data extraction

This pooled study involved searching a range of digital databases, including PubMed, Cochrane Library, Web of Science and Google Scholar, for articles published in English up to May 2015. The study used a subject and text word strategy: ‘breast cancer or breast neoplasms or breast carcinoma or mammary cancer’, ‘CDH13 or cadherin 13 or H-cadherin’, ‘methylation or hypermethylation or epigenetic’. The included articles satisfy the following criteria: (1) original study, and the diagnosis of breast cancer was based on histopathology; table) the subjects in every study included breast cancer patients and normal controls; (3) the articles were focused on methylation of the *CDH13* promoter; (4) the papers were written in English. Exclusion criteria were as follows: (1) the methylation of *CDH13* promoter was conducted only in the cell lines or raw; (2) review papers, commentaries, letters, and studies that examined other associations.

Data were extracted from each study by three independent reviewers (Jingyu Yang and Heng Niu) using pre-specified selection standards. Decisions were made and any disagreements about study selection were resolved by discussion. The following information was extracted from the studies: the first author’s last name, the year of publication, the original country of patients, racial descent (ethnicity), the number of *CDH13* methylations in individual cases and controls in individuals, and more. Racial descent was grouped as Caucasian, Asian, or mixed when ethnic origin was unclear.

### Meta-analysis

The data we acquired were analyzed and visualized mainly using R (R version 3.2.0) software. The strength of the relationship between *CDH13* promoter methylation and the risk/prognosis of breast cancer was measured by a pooled odds ratio (OR)/hazard ratio (HR) with a 95% confidence interval (CI). The significance of the pooled OR was determined by the Z test with a threshold of p<0.05. The I^2^ statistic was used to test the heterogeneity. I^2^ values over 50% and Chi-squared test values of p ≤ 0.1 showed strong heterogeneity between the studies[12]. To explain the heterogeneity of the subgroup differences, we used Tau-squared (τ^2^). The data were pooled using a random effects model (DerSimonian and Laird method) (I^2^>50%, p≤0.1) or fixed effects model (Mantel-Haenszel) (I^2^<50%) according to the heterogeneity statistic I^2^ [13]. When heterogeneity was shown among the included studies, the pooled OR estimates were calculated by the random effects model [14]. Otherwise, the fixed effects model was used [13]. To assess the contributions of single studies to the final results, sensitivity analyses were performed. Generally, the funnel plot symmetry can be used to evaluate the publication bias and whether the results of a meta-analysis were affected by it. Therefore, Begg’s test and Egger’s test were used to examine publication bias [15]. When bias exists, we use a conventional meta-trim method to re-estimate the effect size.

### The extraction and analysis of TCGA data

DNA methylation information for breast cancer was collected from the TCGA project using methylation 450 K datasets [http://cancergenome.nih.gov/]. We calculated the methylation of each CG probe according to the Illumina Infinium Human Methylation 27/450 Bead Array platform instruction: Beta-value = intensity value from the methylated bead type/(intensity values from the methylated + intensity value from unmethylated bead types + 100).

The methylation signals of the 25,978 shared CpG sites in the 450 K datasets were extracted, and the methylation status of each probe was defined according to the beta-value. Any beta value equal to or greater than 0.6 was considered fully methylated. Any beta value equal to or less than 0.2 was considered to be fully unmethylated. Beta values between 0.2 and 0.6 were considered to be partially methylated. The CpG site will be considered methylated when the beta-value is greater than the empirical threshold of 0.3 for tissue/blood data [16].

## Results

### Study characteristics

**Fig 1:**
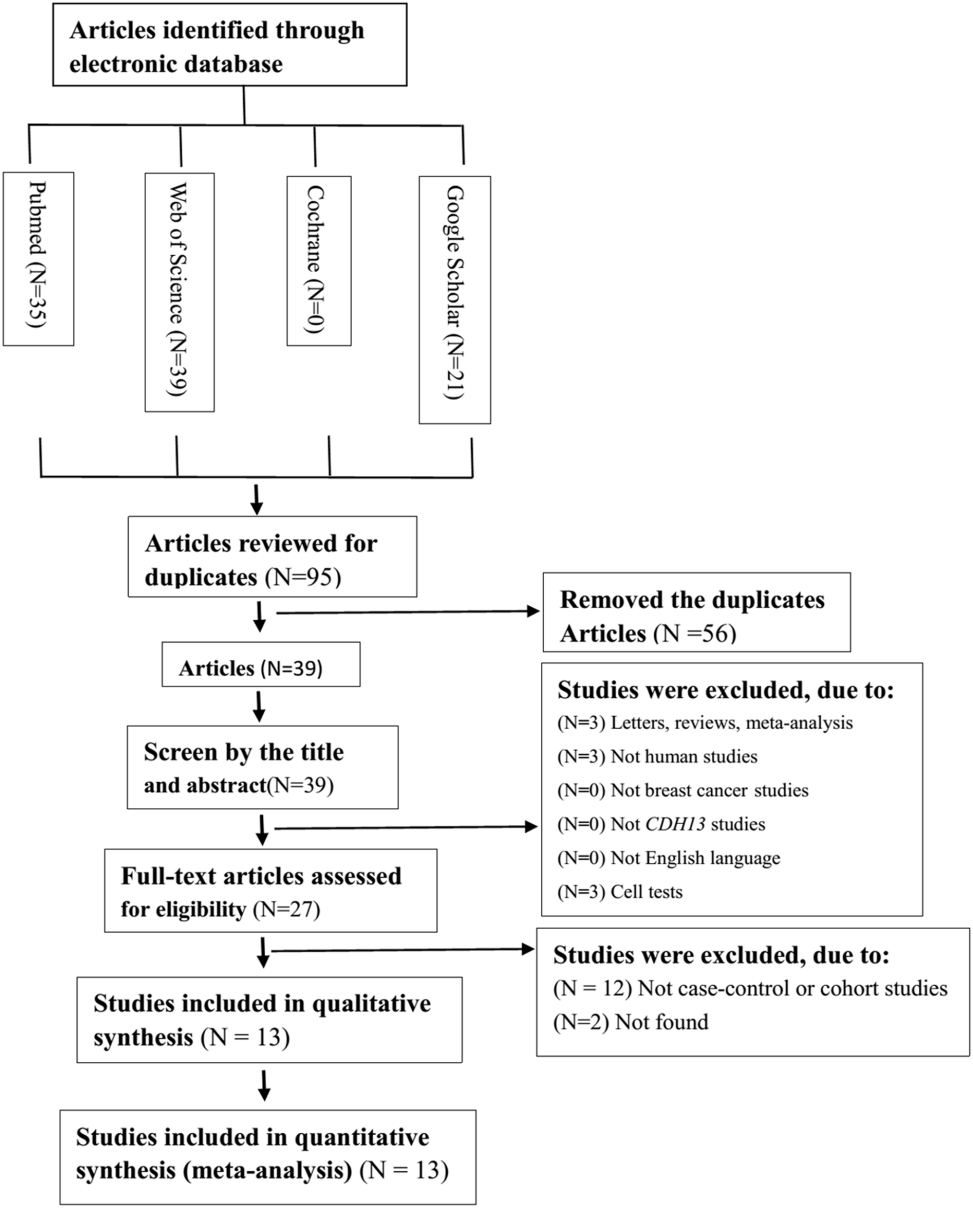
Flow chart of study identification.

Based on the above electronic search strategy, we identified 13 potentially relevant articles (Fig 1) upon further screening for inclusion on the basis of their titles, abstracts and full texts. Finally, 13 articles were selected covering two factors, the risk and prognosis of breast cancer. The characteristics of the 13 articles and the data on risk and prognosis in breast cancer are shown in Tables 1 and 2 [10, 17–29]. All of these articles were written in English. In total, 726 breast cancer tissues/serum samples and 422 normal counterpart tissues/serum samples were collected. The main aim of each study differed; 11 articles were focused predominantly on the risk of breast cancer, whereas the others were focused on prognosis. Among the studies examining the risk of breast cancer, 2 focused on Asian subjects, 6 focused on Caucasian subjects, and 3 examined the risk in mixed-race subjects. We also obtained 3 articles that examined serum and 9 articles that examined tissue. Regarding the experimental methods used to explore *CDH13* promoter methylation status, 5 of 13 inclusions used methylation-specific polymerase chain reaction (MSP), while the others used quantitative MSP (qMSP, such as Methylight, Prosequencing, and so on).

**Table 1:** Characteristics of eligible studies considered in the report.

Author	Year	Country	Method	Sample	Mean age (year)	TNM stage	Male/Female	Control		Case	
								U	M	U	M
Hafez et al.	2015	Kingdom of Saudi Arabia	MSP	Tissue	48.00	I-III	0/180	81	19	53	27
Pang et al.	2014	Australia	MS-HRM	Tissue	54.00	NA	0/80	17	1	37	43
Twelves et al.	2013	UK	MSP	Blood	52.00	I-II	0/54	46	0	42	12
Twelves et al.	2013	UK	MSP	Tissue	52.00	I-II	0/36	46	0	1	35
Jung et al.	2013	Korea	MS-MLPA	Tissue	50.50	I-IV	0/60	57	3	46	14
Wang et al.	2012	USA	BSP	Tissue	54.00	I-III	0/65	65	0	59	6
Verschuur-Maes et al.	2012	Netherlands	MS-MLPA	Tissue	54.00	NA	0/56	9	1	40	16
Kornegoor et al.	2012	Netherlands	MS-MLPA	Tissue	66.00	NA	108/0	10	1	25	83
Moelans et al.	2011	Japan	MS-MLPA	Tissue	NA	NA	NA	7	2	27	8
Chen et al.	2011	USA	MS-MLPA	Blood	NA	NA	NA	12	3	10	7
Feng et al.	2010	Italy	BSP	Tissue	59.00	I-IV	0/76	22	1	10	66
Riener et al.	2008	Germany	Microarray	Blood	55.60	NA	NA	2	0	3	18
Lewis et al.	2005	USA	MSP	Tissue	50.00	0-II	0/38	16	1	24	14

MSP, methylation-specific polymerase chain reaction; MS-HRM, Methylation-sensitive high-resolution melting; MS-MLPA, methylation-specific multiplex ligation-dependent probe amplification; BSP, bisulfite sequencing polymerase chain reaction; M, number of patients with methylation; U, number of patients with unmethylation; NA, not available.

**Table 2:** Baseline characteristics of eligible studies evaluating CDH13 methylation and OS or DFS in breast cancer patients.

Author	Year	Country	Method	Sample	TNM. stage	N	DFS				OS			
							HR	Lower^a^	Upper^a^	p	HR	Lower^a^	Upper^a^	p
Kong et al.	2015	China	IHC	Protein	I-III	142	0.163	0.051	0.52	0.002	0.374	0.11	1.276	0.067
Xu et al.	2012	USA	BSP	DNA	I-II	193	0.75	0.42	1.33	0.32	1.15	0.77	1.7	0.5

N, number of patients; HR: hazard ratios; OS: overall survival; DFS: disease-free survival; BSP, bisulfite sequencing polymerase chain reaction; IHC, immunohistochemistry;

^a^ the upper/lower limit of the 95% confidence interval.

According to the previous study [20], we analyzed 13 different probes located in or near the CDH13 promoter region and chose three of the 13 probes (cg01492639, cg06341397, cg02263260) containing the transcription start site of the CDH13 gene. A total of 699 breast cancer tissue/blood samples and 96 normal samples were obtained from the TCGA project (S1 Table). Of the 699 patients, 18.60% had CDH13 methylation, while there was no methylation of *CDH13* in the normal sample. Among the 699 patients, there were 677/7 female/male. The patients’ ages ranged from 26 to 90, and the AJCC pathologic tumor stage ranged from I-X.

### Meta-analysis of risk and prognosis in breast cancer

**Fig 2:**
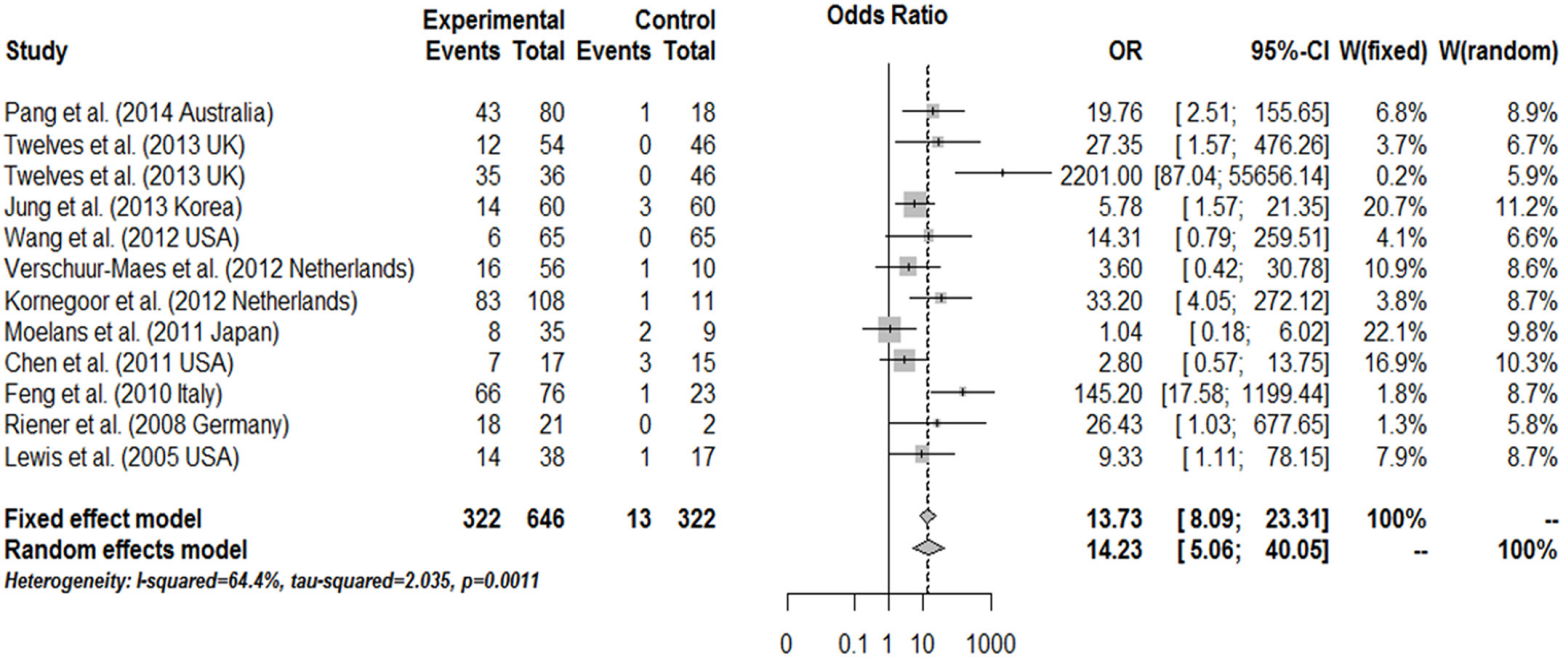
Combined estimates for the association between *CDH13* promoter methylation and breast cancer with forest plot. Author, year, and country of the studies, methylated (M) and total number of samples (T) in case and control, and combined odds ratio (OR) with 95% confidence region were labeled in the right column of the figure. The DerSimonian-Laird estimator and Mantel-Haenszel method were selected to conduct combination estimation for the random effects model and fixed effects model, respectively.

The results of the association between the promoter methylation of *CDH13* and breast cancer are shown in Fig 2. For the *CDH13* gene association with breast cancer risk, our findings demonstrate that the frequency of *CDH13* promoter methylation in cancer was OR = 13.73, 95%CI: 8.09~23.31, z = 9.70, p<0.0001 by the fixed effect model and OR = 14.23, 95%CI: 5.06~40.05, z = 5.031, p<0.0001 by the random effect model, which suggests a statistically significant increase in the likelihood of *CDH13* promoter methylation in breast cancer compared to the controls. Upon analyzing the prognosis data for *CDH13* promoter methylation in breast cancer patients, we found that the HR was 0.77 (95%CI: 0.27~2.21, z = -0.49, p = 0.622) and 0.38 (95%CI: 0.09~1.69, z = -1.27, p = 0.20) for overall survival (OS) and disease-free survival (DFS), respectively, as assessed by the random effect model with any heterogeneity. This result indicates that breast cancer patients with *CDH13* promoter methylation have limited prognostic value for clinical diagnosis.

**Fig 3:**
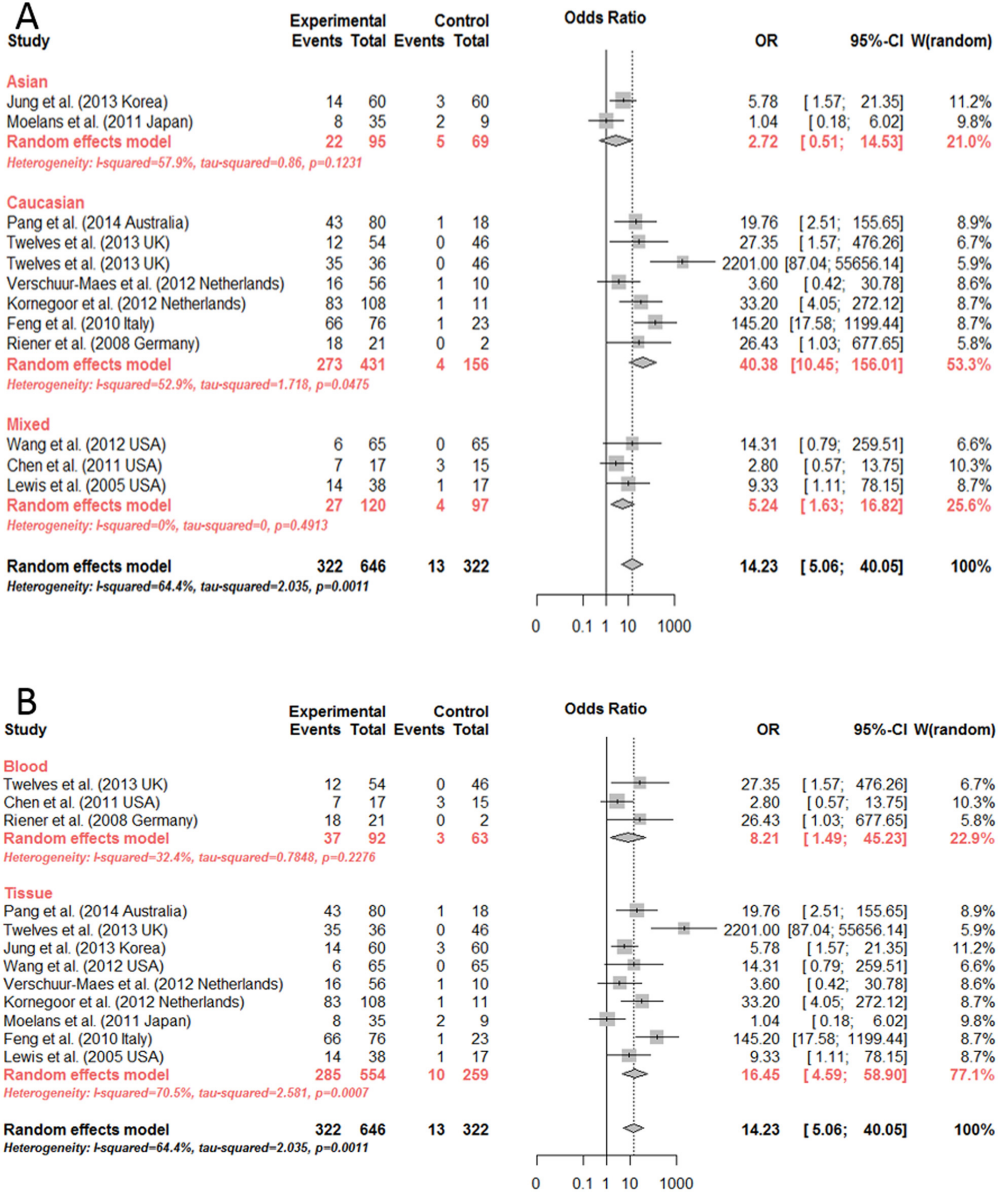
Subgroup meta-analysis for the relationship between *CDH13* promoter methylation and breast cancer. (A, B). Subgroup meta-analysis based on race and sample by random effects model and fixed effects model, respectively.

Significant differences were found among the mixed-race (OR = 5.24, 95%CI: 1.63~16.82, by random effects model), Caucasian (OR = 40.38, 95%CI: 10.45~156.01, by random effects model) and Asian (OR = 2.72, 95%CI: 0.51~14.53, by fixed effect model) subgroups (p<0.0001) (Fig 3A). As we have inferred, studies of both tissue and serum showed a significant association between *CDH13* methylation and breast cancer (OR = 16.45, 8.21 by random effects model, respectively) (Fig 3B). Hence, the methylation status of *CDH13* could be used as a potential biomarker for breast cancer diagnosis for either tissue or serum samples.

### Bias analysis and sensitivity analysis

**Fig 4:**
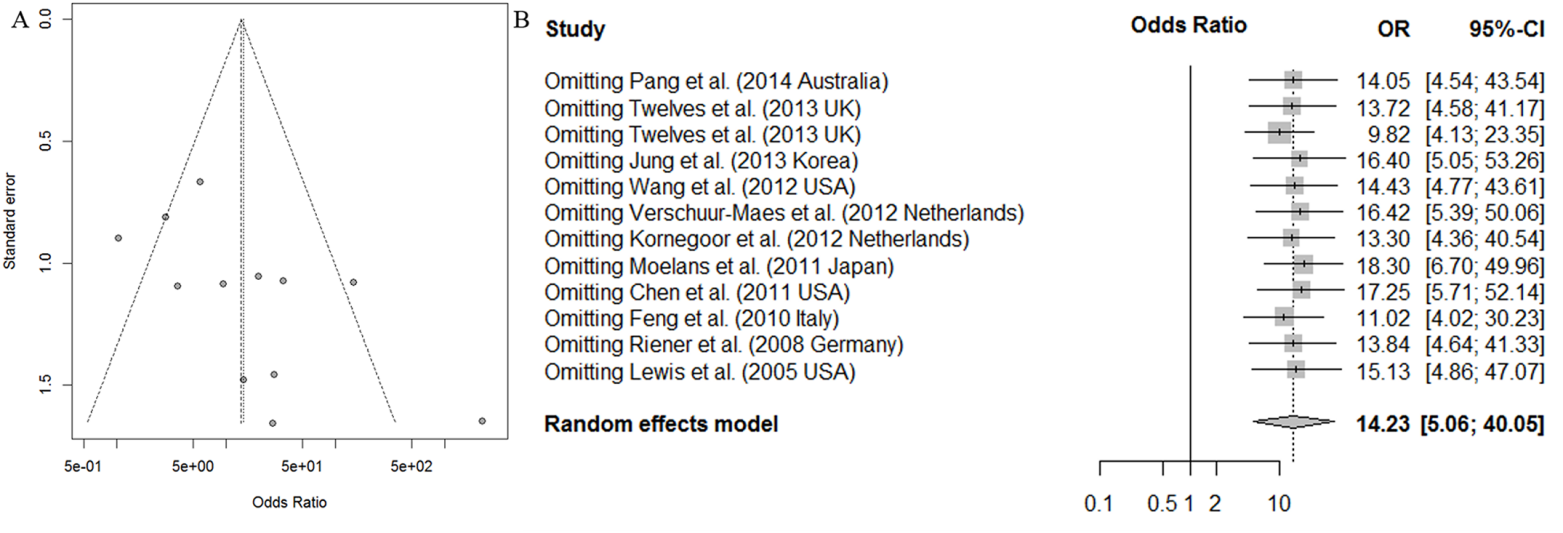
Funnel plot for publication bias test and sensitivity analysis of the summary odds ratio coefficients on the relationships between *CDH13* methylation and breast cancer patients.

To assess the publication bias of the articles, we used Begg’s test and Egger’s test. The assessment of Egger’s test revealed some evidence of obvious asymmetry in the ensemble analysis (t = -1.11, p = 0.29), and Begg’s test indicated an absence of publication bias (t = 2.43, p = 0.035) (Fig 4A).

To our knowledge, the modification of the inclusive criteria of the meta-analysis may affect the final results. Therefore, a sensitivity analysis was conducted. The OR of the sensitivity analysis ranged from 10.21 (95% CI, 5.82~17.91) to 15.95 (95% CI, 8.94~28.44) upon omitting a single study with the random effect model (Fig 4B). Hence, as indicated by the sensitivity analysis, no single study would affect the pooled OR.

### Validation by independent TCGA breast cancer dataset

To validate the results of the meta-analysis, we collected the data on the *CDH13* methylation status, and clinical characteristics from the breast cancer samples of the TCGA project. We then separated the data into several subgroups based on the meta-analysis.

**Fig 5:**
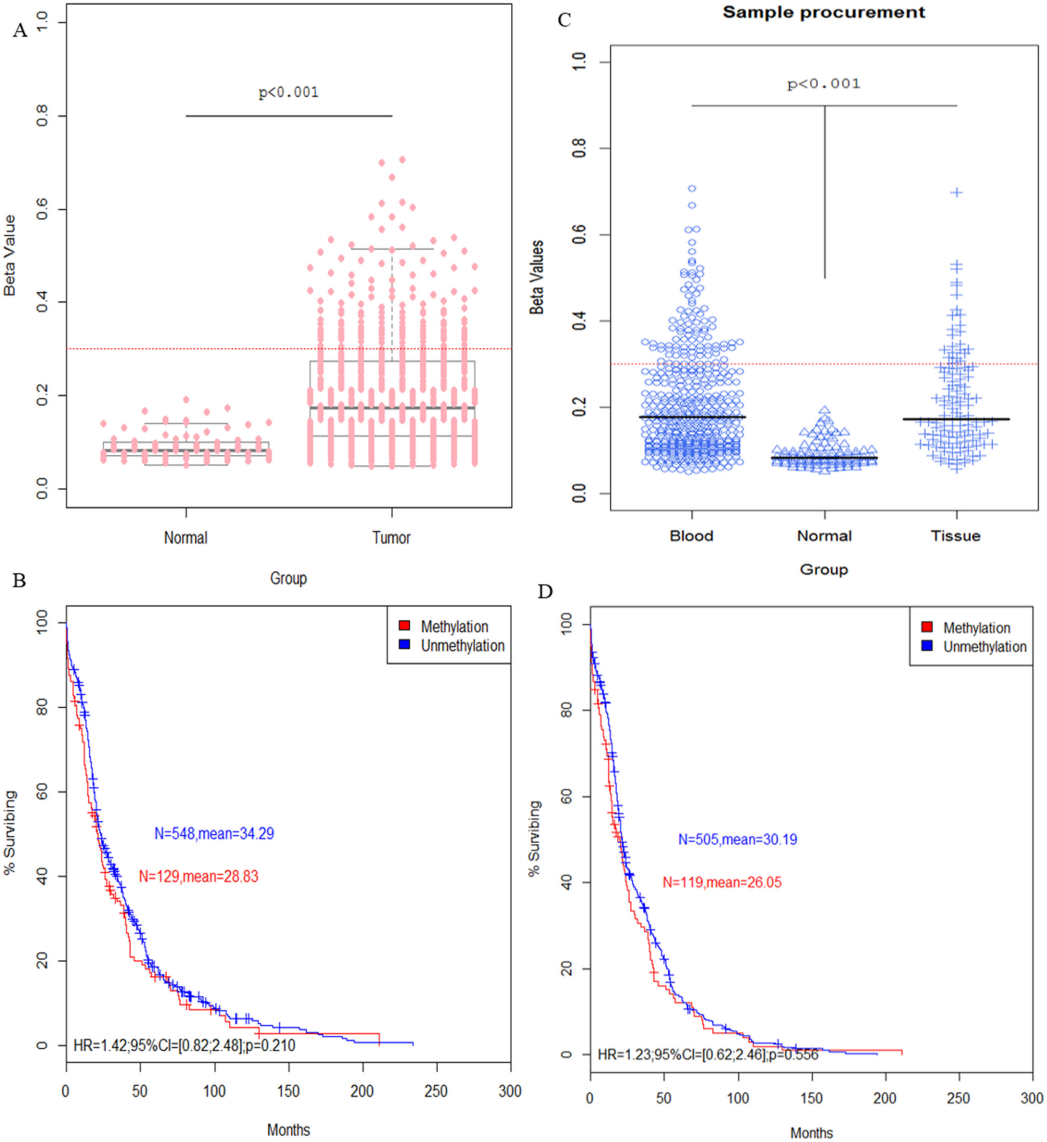
The relationships of *CDH13* methylation with TCGA probe, sample source and survival curve of breast cancer in the TCGA data. (A). Different TCGA probe for 450 K datasets shows the relationship of *CDH13* methylation and breast cancer risk (N_Tumor_ = 699, N_Normal_ = 96, respectively). (C). The *t*-test indicates significant differences in blood (N = 444) and tissue (N = 120) samples compared to normal tissue (N = 96). (B, D). Association of patient survival and *CDH13* methylation status by Kaplan-Meier method. Red dotted line indicates β = 0.3.

**Fig 6:**
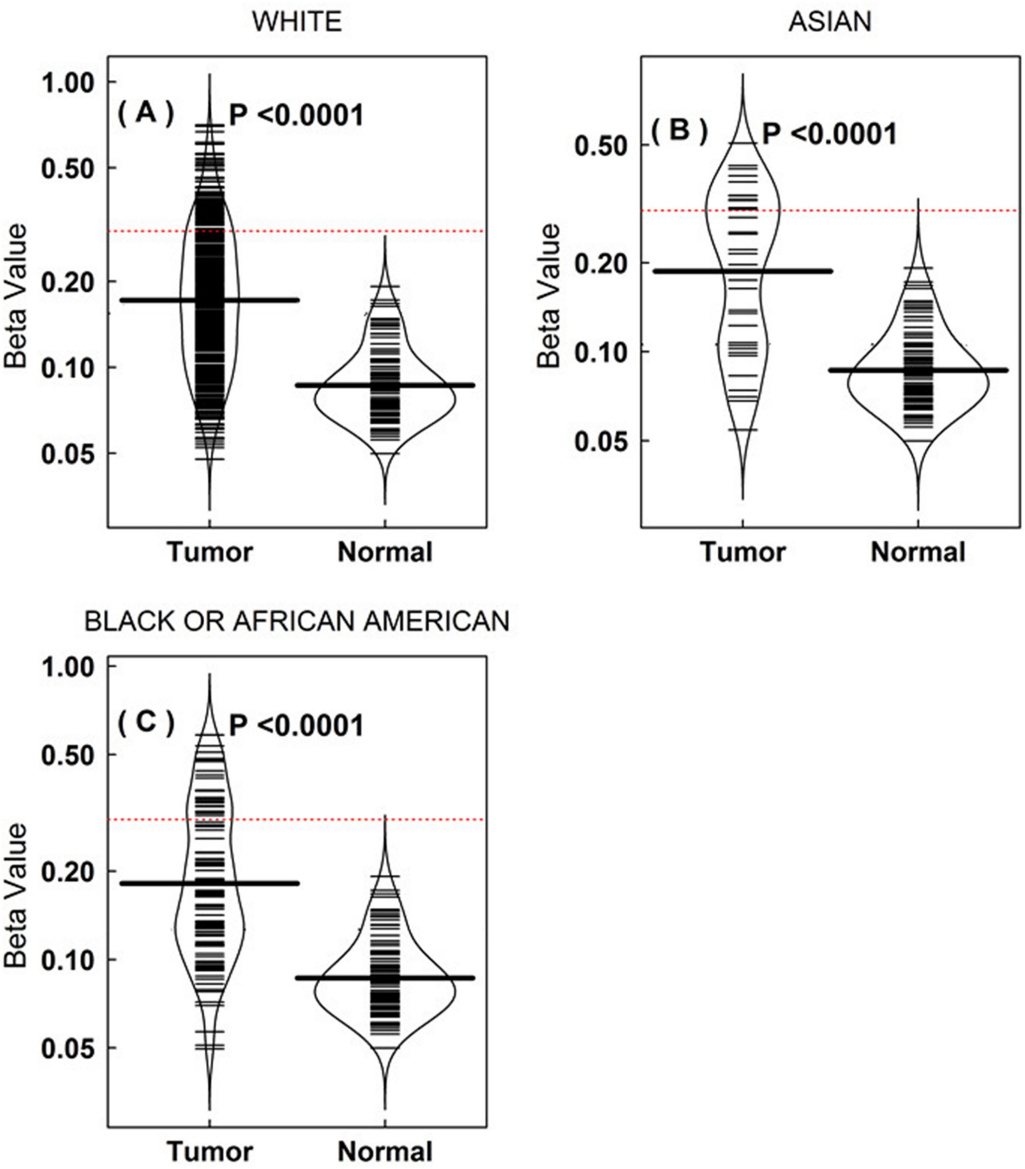
Different racial types affecting the OR of the *CDH13* methylation to the risk of breast cancer in the TCGA data. (A-C). The beanplot demonstrates that different racial types affect the *CDH13* methylation to the risk of breast cancer in data from TCGA breast cancer datasets. The number of tumor patients is 524, 34, 101 in White, Asian and Black or African American people, respectively. Black dotted line indicates β = 0.3. The differences are significant by *t*-test.

In the DNA methylation microarray dataset from the TCGA project, there was a significant difference between the cancer cases and controls (Fig 5A); the same result was also found in the race subgroup (Fig 6). According to the t-test, both the tissue resection and blood draw had greater significance than the control groups (Fig 5C).

To date, the potential of gene-specific DNA methylation has been reported as a prognostic indicator in many cancers [30–36]. In this study, we set out to explore the relationship between *CDH13* promoter methylation and breast cancer survival using data extracted from the TCGA project. As predicted, patients without *CDH13* methylation live almost the same time as patients with *CDH13* methylation (HR = 1.41, 95% CI = [0.82; 2.48], p = 0.210) (Fig 5D), suggesting that *CDH13* methylation does not decrease the survival rate in patients with breast cancer. DFS can also demonstrate a relationship to the prognosis, and the result (HR = 1.23, 95% CI = [0.62; 2.46], p = 0.556) is similar to the OS (Fig 5B). The same result was found using our meta-analysis.

## Discussion

Alterations to DNA promoter methylation are the most frequent molecular changes associated with many human cancers [37–39]. Aberrant promoter methylation has been described for many different genes in various malignancies, suggesting that specific tumors might have their own distinct methylation profiles [38, 40]. The traditional diagnosis of malignant tumors uses microscopy to check for morphological changes, based on visible histopathological differences between tumor and normal tissue. Since 2000, the World Health Organization (WHO) made some major additions to the tumor classification criteria. Comprehensive information was added, such as the immune phenotype, genetic characteristics, clinical manifestation, and imaging to define tissue pathology, both sorted and graded. Compared to ordinary histopathological diagnosis, the additional information is now more beneficial, as it allows the individualized treatment of a given tumor. Hence, oncogene and tumor suppressor genes could be used as a new generation of detection markers in the clinical diagnosis of tumors. In recent years, a growing number of studies have shown that both the occurrence and development of many different tumors are linked to abnormal DNA methylation, and the abnormal methylation of tumor specific genes can be detected early, even before clinical diagnosis. Such analysis of DNA methylation offers several advantages, as it can be detected in fluids such as serum. Given that *CDH13* promoter methylation is the most common genetic inactivation event found in human tumors, it would be a useful marker for the early detection of cancer.

Recent studies have reported that *CDH13* is an important tumor suppressor in breast cancer patients [18, 41, 42]. Consequently, many researchers have tried to explore the role of *CDH13* methylation and the epigenetics of other genes in the prognosis and early detection of breast cancer, as well as the differentiation between malignant and non-malignant lesions. Here, we conducted a meta-analysis to explore the association between *CDH13* methylation and breast cancer. The systematic literature search yielded a total of 13 studies, comprising 11 studies of breast cancer risk and 2 articles on breast cancer prognosis, which were used for the final analysis. Our results revealed that the frequency of *CDH13* promoter methylation in breast cancer tissue/serum was significantly higher than in normal tissue/serum. This finding was true in patients of African, Asian and Caucasian descent, suggesting a statistically significant increase in the likelihood of methylation in breast cancer compared to the controls. However, *CDH13* promoter methylation was not significantly related to the OS and DFS of breast cancer. Therefore, *CDH13* promoter methylation is likely to be linked to the risk of breast cancer and may have limited prognostic value for breast cancer patients.

To our knowledge, heterogeneity would affect the credibility of the meta-analysis result. As shown by the meta-analysis, there is some heterogeneity in the risk (tau^2^ = 2.04, I^2^ = 64.4%, p = 0.001) and DFS ((tau^2^ = 0.95, I^2^ = 81.2%, p = 0.021)) of breast cancer. Although sensitivity analyses found that there was no single sensitive study in this meta-analysis, Begg’s and Egger’s tests demonstrated no evidence of publication bias; we took data from the TCGA project, which would provide an additional resource that might be without publication bias, to prove the meta-analysis result. As predicted, the same result was found in the data of TCGA project of *CDH13* methylation in breast cancer. Taken together, the results of this systematic review indicate a credible relationship between *CDH13* methylation and breast cancer.

In conclusion, this meta-analysis of the article data provides strong evidence that the methylation status of the *CDH13* promoter is strongly related to breast cancer risk. However, *CDH13* methylation is not prognostic for breast cancer patients. Future studies are needed that collectively explore the possible roles of *CDH13* methylation in breast cancer.

## Supporting Information

S1 PRISMA Checklist PRISMA Checklist. (DOCX)

S1 Table Characteristics of the data obtained from the TCGA project. (XLSX)
