# Supplementary material for: A Systematic Analysis of the Relationship of CDH13 Promoter Methylation and Breast Cancer Risk and Prognosis
Source: PLoS One. 2016 May 6;11(5):e0149185. doi: 10.1371/journal.pone.0149185 (PMC4859545; doi:10.1371/journal.pone.0149185)
Supplement: S1 PRISMA Checklist — (DOCX) [file pone.0149185.s001.docx]

| Section/Topic | # | Checklist item | Reported on Page # |
| --- | --- | --- | --- |
| TITLE | | | |
| Title | 1 | Indicate the study design with a commonly used term in the title. | 1 |
| ABSTRACT | | | |
| Abstract | 2 | Provide a structured summary including, as applicable: background; objectives; data sources; synthesis methods; results; conclusions and implications of key findings. | 2 |
| INTRODUCTION | | | |
| Background | 3 | Include sufficient scientific background (including relevant references to previous work) to understand the motivation and context for the study. | 3 |
| Objectives | 4 | Provide an explicit statement of questions being addressed with reference to participants, interventions, comparisons, outcomes, and study design. | 3-4 |
| METHODS | | | |
| Eligibility criteria | 5 | Specify study characteristics and report characteristics (e.g., years considered, language, publication status) used as criteria for eligibility, giving rationale. | 4 |
| Information sources | 6 | Describe all information sources (e.g., databases with dates of coverage, contact with study authors to identify additional studies) in the search and date last searched. | 4/6 |
| Search | 7 | Present full electronic search strategy for at least one database, including any limits used, such that it could be repeated. | 4 |
| Study selection | 8 | State the process for selecting studies (i.e., screening, eligibility, included in systematic review, and, if applicable, included in the meta‐analysis). | 4/6 |
| Data collection process | 9 | Describe method of data extraction from reports (e.g., piloted forms, independently, in duplicate) and any processes for obtaining and confirming data from investigators. | 4-5 |
| Data items | 10 | List and define all variables for which data were sought and any assumptions and simplifications made. | 4 |
| Risk of bias in individual studies | 11 | Describe methods used for assessing risk of bias of individual studies. | 5-6 |
| Summary measures | 12 | State the principal summary measures (e.g., risk ratio, difference in means). | 5 |
| Synthesis of results | 13 | Describe the methods of handling data and combining results of studies, if done, including measures of consistency (e.g., I^2^) for each meta‐analysis. | 5 |
| Risk of bias across studies | 14 | Specify any assessment of risk of bias that may affect the cumulative evidence (e.g., publication bias, selective reporting within studies). | 5 |
| Additional analyses | 15 | Describe methods of additional analyses (e.g., sensitivity or subgroup analyses, meta-regression), if done, indicating which were pre‐specified. | 5 |
| RESULTS | | | |
| Study selection | 16 | Give numbers of studies screened, assessed for eligibility, and included in the review, with reasons for exclusions at each stage, ideally with a flow diagram. | 6-7 |
| Study characteristics | 17 | For each study, present characteristics for which data were extracted (e.g., study size, PICOS, follow-up period) and provide the citations. | 6-7 |
| Risk of bias within studies | 18 | Present data on risk of bias of each study and, if available, any outcome level assessment | 6-7 |
| Results | 19 | For all outcomes considered (benefits or harms), present, for each study: (a) simple summary data for each intervention group (b) effect estimates and confidence intervals, ideally with a forest plot (c) the result of TCGA dataset | 7-9 |
| Synthesis of results | 20 | Present results of each meta-analysis done, including confidence intervals and measures of consistency. | 8-9 |
| Risk of bias across studies | 21 | Present results of any assessment of risk of bias across studies | 9 |
| Additional analysis | 22 | Give results of additional analyses, if done (e.g., sensitivity or subgroup analyses) | 7 |
| DISCUSSION | | | |
| Summary of evidence | 23 | Summarize the main findings including the strength of evidence for each main outcome. | 10-11 |
| Interpretation | 24 | Give a cautious overall interpretation of results considering objectives, multiplicity of analyses, results from similar studies, and other relevant evidence | 11 |
| Significance | 25 | Discuss the significance (external validity) of the study results | 11 |
| Conclusions | 26 | Provide a general interpretation of the results in the context of other evidence, and implications for future research. | 11-12 |
| FUNDING | | | |
| Funding | 27 | Give the source of funding and the role of the funders for the present study and, if applicable, for the original study on which the present article is based | 12 |
